# Etymologia: *Staphylococcus*

**DOI:** 10.3201/eid1909.ET1909

**Published:** 2013-09

**Authors:** Giancarlo Licitra

**Keywords:** etymologia, *Staphylococcus*, bacteria, gram-positive spherical bacteria

## *Staphylococcus* [staffʺə-lo kokʹəs]

From the Greek *staphyle* (bunch of grapes) and *kokkos* (berry), *Staphylococcus* is a genus of gram-positive spherical bacteria that commonly cause surgical and skin infections, respiratory disease, and food poisoning. In 1880, Scottish surgeon Sir Alexander Ogston first described staphylococci in pus from a surgical abscess in a knee joint: “the masses looked like bunches of grapes.” In 1884, German physician Friedrich Julius Rosenbach differentiated the bacteria by the color of their colonies: *S. aureus* (from the Latin *aurum*, gold) and *S. albus* (Latin for white). *S. albus* was later renamed *S. epidermidis* because of its ubiquity on human skin.
